# Psychological Support in Palliative Care: Findings from a Retrospective Single Centre Cross-Sectional Study in Slovenia

**DOI:** 10.1192/j.eurpsy.2025.1790

**Published:** 2025-08-26

**Authors:** A. Rojs, L. Ternar, R. Globevnik, M. Horvat, A. Žist

**Affiliations:** 1 Maribor University Medical Centre, Maribor, Slovenia; 2 Department of Oncology, Maribor University Medical Centre, Maribor, Slovenia

## Abstract

**Introduction:**

Common psychiatric disorders in oncological patients include depression, anxiety, delirium, sexual dysfunctions, adjustment and sleep disorders, affecting 30%–40% of those diagnosed. The incidence is even higher in advanced cancer stages. Despite the profound impact on quality of life, these issues are often underdiagnosed and undertreated. While effective management includes both pharmacological and nonpharmacological interventions, psychologists are rarely involved in palliative care, as doctors tend to focus primarily on physical symptoms overlooking the emotional and existential suffering these patients endure.

**Objectives:**

The aim of this study was to determine the frequency of clinical psychologist involvement in the care of palliative cancer patients.

**Methods:**

We performed a single centre cross-sectional study of patients with metastatic or locally advanced, inoperable cancer who were hospitalized at the University Clinical Centre Maribor every first Wednesday of October between 2017 and 2023. Patient records were retrieved from the hospital’s electronic medical database. Data extraction included demographic information, clinical details, and whether a clinical psychologist was consulted by the treating physician. We also aimed to identify the reasons for consultation when applicable.

**Results:**

Out of the 600 patients, 342 (57.0%) were male, and 258 (43.0%) were female. Their ages ranged from 30 to 95 years, with a mean age of 67.9 ± 11.1 years and a median age of 68 years. Only 1.3% (8 patients) had a clinical psychologist involved in their care through a doctor-initiated consultation while most (592 patients or 98.7%) did not. Anxiety-related concerns were the most common reasons for consulting a psychologist, with 4 patients addressing these issues: 1 patient expressed a fear of dying, another feared the pain, a third had concerns about potential side effects of therapy, especially infertility, and the fourth presented general anxiety. Additionally, 1 patient expressed suicidal ideations. Unfortunately, the reason for consulting was not available for 3 patients.

**Conclusions:**

Our study shows the underutilization of clinical psychologists in the care of palliative cancer patients, with only a small part receiving psychological support despite the known prevalence of psychiatric disorders in this population. Given the comorbidity of depression, anxiety and other psychiatric disorders among these patients, integrating mental health professionals into palliative care teams is crucial for enhancing their overall quality of life and emotional well-being.

**Disclosure of Interest:**

None Declared

